# Community-Based Research as a Mechanism to Reduce Environmental Health Disparities in American Indian and Alaska Native Communities

**DOI:** 10.3390/ijerph120404076

**Published:** 2015-04-13

**Authors:** Cynthia Agumanu McOliver, Anne K. Camper, John T. Doyle, Margaret J. Eggers, Tim E. Ford, Mary Ann Lila, James Berner, Larry Campbell, Jamie Donatuto

**Affiliations:** 1 Office of Research and Development, National Center for Environmental Research, United States Environmental Protection Agency, 1200 Pennsylvania Avenue, N.W., Washington, DC 20460, USA; 2 College of Engineering, Montana State University, Bozeman, MT 59717, USA; E-Mail: anne_c@erc.montana.edu; 3 Apsaalooke Water and Wastewater Authority, Crow Environmental Health Steering Committee/Little Big Horn College, Crow Agency, MT 59022, USA; E-Mail: doylej@lbhc.edu; 4 Center for Biofilm Engineering, Montana State University, Bozeman, MT 59717, USA; E-Mail: mari.eggers@biofilm.montana.edu; 5 School of Health Professions, Shenandoah University, 1460 University Drive, Winchester, VA 22601, USA; E-Mail: tford@su.edu; 6 Plants for Human Health Institute, North Carolina State University, N.C. Research Campus, 600 Laureate Way, Kannapolis, NC 28081, USA; E-Mail: mlila@ncsu.edu; 7 Alaska Native Tribal Health Consortium, 3900 Ambassador Drive, Anchorage, AK 99508, USA; E-Mail: jberner@anthc.org; 8 Tribal Historic Preservation Officer, Swinomish Indian Tribal Community, 11430 Moorage Way, La Conner, WA 98257, USA; E-Mail: lcampbell@swinomish.nsn.us; 9 Environmental Health Analyst, Department of Social Services, Swinomish Indian Tribal Community, 17337 Reservation Road, La Conner, WA 98257, USA; E-Mail: jdonatuto@swinomish.nsn.us

**Keywords:** American Indian and Alaska Native (AIAN), tribal communities, Community-Based Participatory Research (CBPR)

## Abstract

Racial and ethnic minority communities, including American Indian and Alaska Natives, have been disproportionately impacted by environmental pollution and contamination. This includes siting and location of point sources of pollution, legacies of contamination of drinking and recreational water, and mining, military and agricultural impacts. As a result, both quantity and quality of culturally important subsistence resources are diminished, contributing to poor nutrition and obesity, and overall reductions in quality of life and life expectancy. Climate change is adding to these impacts on Native American communities, variably causing drought, increased flooding and forced relocation affecting tribal water resources, traditional foods, forests and forest resources, and tribal health. This article will highlight several extramural research projects supported by the United States Environmental Protection Agency (USEPA) Science to Achieve Results (STAR) tribal environmental research grants as a mechanism to address the environmental health inequities and disparities faced by tribal communities. The tribal research portfolio has focused on addressing tribal environmental health risks through community based participatory research. Specifically, the STAR research program was developed under the premise that tribal populations may be at an increased risk for environmentally-induced diseases as a result of unique subsistence and traditional practices of the tribes and Alaska Native villages, community activities, occupations and customs, and/or environmental releases that significantly and disproportionately impact tribal lands. Through a series of case studies, this article will demonstrate how grantees—tribal community leaders and members and academic collaborators—have been addressing these complex environmental concerns by developing capacity, expertise and tools through community-engaged research.

## 1. Introduction

American Indian and Alaska Native peoples and communities (AIAN) are faced with ongoing environmental health challenges that demand collaborative and sustained research, innovative methods, and culturally appropriate interventions. Climate change is adding to these impacts on Native American communities [1], variably causing drought, increased flooding and forced relocation [2], affecting tribal water resources [3], traditional foods [4,5], forests and forest resources [6], and tribal health [7,8]. This article will highlight several extramural research projects supported by the United States Environmental Protection Agency (USEPA) Science to Achieve Results (STAR) tribal environmental research grants as a mechanism to address the environmental health inequities and disparities faced by tribal communities [9].Ongoing environmental health issues facing AIAN communities include legacies of environmental pollution and contamination [10,11], limited access to clean water [12,13], diminishing quantity and quality of culturally important natural resources, including subsistence foods summarized in [14], which has many consequences including diminished engagement in the practice of subsistence, reliance on non-indigenous food diets and the increased prevalence of glucose intolerance and Type 2 Diabetes [15]. All of these challenges may be further exacerbated by the impacts of climate change (*cf.* [1,2,4,7,8]). Recent and on-going AIAN-led and AIAN-partner based research is making headway in clearly linking environmental health problems to health outcomes and crafting interventions amidst the complexity of the hazard-effects continuum.

It is well documented that AIAN communities are disproportionately impacted by diabetes, respiratory diseases, liver disease, cancers, cardiovascular diseases, unintentional injuries, and suicide [16], as well as increased rates of infant mortality and sexually transmitted diseases [17]. The root causes of these health outcomes are the myriad inequities that challenge maintaining and sustaining health—colonial oppression, generations of trauma, and racism (*cf.* [18]). Some of the many effects include*:* a legacy of economic adversity and poor social conditions, inadequate education, disproportionate poverty, discrimination in the delivery of health services, and cultural barriers [16,19]. The end result for AIAN communities is an overall lower life expectancy and decreased quality of life, compared to other racial/ethnic populations.

The impacts of environmental problems add to health disparities in ways both obvious and subtle; these health effects are often difficult to tease out among the numerous other causes. For instance, AIAN communities and many communities of color live in areas that are disproportionately co-located with environmental hazards such as nuclear test sites, uranium mines, power plants, toxic waste dumps and other sources of pollution [20,21,22,23,24]. While the issue with geographic proximity is clear, and linking proximity to health impacts is intuitive, often it is difficult to establish cause–effect relationships due to the presence of many other health inequities and the often chronic nature of exposures. Even more difficult is when the effects are subtle, or “invisible,” and elude typical methods, such as when culturally important resources are polluted and the deeply rooted interconnections between people and the natural world (social, cultural and spiritual) are also impacted, which cannot be measured with conventional quantitative assessment methods [25,26]. These impacts cannot be evaluated with conventional tools such as human health risk assessments because these methods focus only on individual, physiological health and are not based on community-driven health definitions and priorities. Individual, physiological health parameters simply are too narrow to reflect the many connections between community health and first foods (also called traditional foods, subsistence foods or country foods) [27,28].

In order to effectively address AIAN environmental health disparities, several key conditions must be met: (1) *Cultural relevance.* AIAN communities define their health priorities themselves, as each community is unique and even neighboring communities may have very different health concerns. AIAN communities drive the research design, implementation and dissemination of results to ensure relevance to community, and are meaningfully engaged throughout the process if they are partnering with academic or other institutions; (2) *Mutual respect and trust.* AIAN communities and their collaborators and funders need to establish, develop and nurture respect and trust; (3) *Adequate and sustained resources*. Long term, sustaining resources are necessary for AIAN communities to evaluate and enact long-term health interventions; and, (4) *Sustainable Partnerships.* For AIAN communities who chose to partner, sustained committed relationships with academic or other research partners must be established and maintained past the completion of one project. These key conditions are often sought after by AIAN communities and partners via grants from federal agencies via the use of a Community-Based Participatory Research (CBPR) model. CBPR is a collaborative approach that equitably involves partners in the entire research process—from formulation of the project purpose and questions through outputs and outcomes—and in which all partners contribute expertise, share decision making and ownership [29,30]. While many researchers have put forth models of CBPR, our conceptual model of the four key conditions and their connection to CBPR is reflected in the conceptual model from Wallerstein *et al.* [31,32]).

This paper highlights five projects either led by AIAN communities or by partnerships between AIAN communities and academic/research institutions, all of which have been funded via the U.S. Environmental Protection Agency’s (EPA) National Center of Environmental Research (NCER) Science to Achieve Results (STAR) Program. The STAR program’s “Tribal Research” portfolio [9] addresses AIAN environmental health risks through tribally engaged research with the goal of creating culturally specific, effective interventions. Initiated in 1999, the STAR research program was developed under the premise that AIAN populations may be at an increased risk for environmentally-induced diseases as a result of unique lifestyle practices, community activities, occupations and customs, and/or environmental releases that significantly and disproportionately impact AIAN lands. The STAR portfolio has supported three solicitations and 10 grants valued at $6 Million exploring how cumulative chemical exposures and global climate change are affecting Tribes, and to better understand the health effects of environmental contaminants on Tribal populations (Link to the solicitations may be found on the EPA’s webpage: www.epa.gov/ncer/rfa/archive and the STAR funded grant projects (with contact information for the AIAN communities and their partners) can be found at: www.epa.gov/ncer/tribalresearch and http://www.epa.gov/ncer/tribalresearch/recipients.html).

The research conducted by the grantees and their partners highlighted in this paper illustrates how AIAN communities identify solutions and apply interventions that have reduced negative health and ecological effects from the consumption of water and water-based food resources, other exposures to chemical contaminants, and impacts of climate change, while enhancing their ability for community-level risk assessments. A synopsis of each project is provided in the next section. All of these projects demonstrate the use of CBPR practices to meet the first three of the four key conditions: cultural relevancy, mutual respect and trust, and adequate and sustained resources. The fourth key condition, sustained partnerships, is on-going, and described in each case study. The authors consider all four key conditions as critically important to achieve successful and sustained community research endeavors; efforts to achieve the key conditions are summarized by case study in Table 1.

## 2. Case Studies of STAR Tribal Environmental Research Projects

### 2.1. Swinomish Indian Tribal Community

PI and Senior Personnel: Jamie Donatuto, PI for the STAR project, Environmental Health Analyst, Swinomish Indian Tribal Community; Larry Campbell, Senior Researcher, Swinomish Elder and Tribal Historic Preservation Officer, Swinomish Indian Tribal Community.

**Table 1:** Important factors identified in case studies for successful and sustained community-based research.

Studies	Cultural Relevance	Mutual Respect and Trust	Adequate and Sustained Resources	Sustainable Partnerships
*Swinomish Indian Tribal Community: Indigenous Health Indicators*	Swinomish focused on the lack of tribal-specific health indicators in health assessments with the goal of improving how tribal health is evaluated and addressed for themselves and Tribes.	Swinomish designed and enacted the study with STAR funds awarded directly to the Tribe.	The funding provided additional time and resources needed to enact the identified research that was additional to the Tribe’s established programs and operations.	Four other Coast Salish Tribes partnered with Swinomish to refine and pilot-test the Indigenous Health Indicators, strengthening research relationships between the Tribes.
*Apsaalooke (Crow Tribe): Environmental Health—Water Quality*	Apsaalooke people identified contaminated water as their greatest environmental health concern.	The Crow Environmental Health Steering Committee, composed of Tribal stakeholders, recruited academic partners, and initiated, guided and set the priorities for our work.	Federal and state funding (for infrastructure) continues to flow directly to the tribe, in part as a result of compelling data from our studies. New funding is helping with intervention research.	The partnership between the Apsaalooke people, Little Big Horn College and MSU continues to build—with an increasing focus on solutions to environmental health needs and pipelines into college, through graduate education.
*Alaska Native Tribal Communities: Wild Berry Resources*	AN communities were concerned that climate change may alter the traditional medicinal value and/or availability of indigenous berry resources.	Community councils from Akutan, Seldovia, and Point Hope held multiple community forums to discuss outcomes with project team and to craft synopsis for Tribal publications.	Funding was sufficient to equip each AN community with bioassay kits for up to 2 years of follow up work in local schools. Subsequent USDA funding was obtained to aid in science curriculum development using Native resources.	Partnerships continue with UI, NCSU and Rutgers with ANTHC (Alaska Native Tribal Health Consortium) and local school leaders in AN communities.
*Yupik Alaska Native Maternal Biomonitoring Program: Assessing Food Safety and Adaptive Strategies*	Yupik residents of southwestern Alaska requested an investigation of risks and benefits of their traditional diet, and an investigation of maternal and infant contaminant and micronutrient exposure.	The tribal organizations designed their own study, and applied for EPA funding, which was initially awarded in an interagency agreement with the IHS, and subsequently with a STAR grant.	The funding was supplemented with tribal organization funding, and together, the funding was adequate.	The Alaska Native Tribal Health Consortium is actively planning a long-term maternal monitoring program, with methodological modifications to reduce cost.

The discussion will touch on next steps forward for the other key conditions—the long-term sustained resources and relationships crucial for building and maintaining capacity in AIAN communities.

#### 2.1.1. Background

The Swinomish Indian Tribal Community is a federally recognized tribe in the Pacific Northwest United States. The Swinomish received a U.S. EPA STAR grant to create, pilot test, and evaluate a set of environmental public health indicators that reflect the Coast Salish (Tribal) communities’ meanings and prioritizations of health (“Tribal Environmental Public Health Indicators”, RD#83479101, 2011–2014). The project was the logical continuation of over a decade of work initiated by the Swinomish due to the lack of health indicators available that accurately depict tribal health. For more than 20 years, tribes have been requesting that tribal-specific definitions of health be equitably employed in health risk assessments; yet to date, no alternatives have been accepted or incorporated into the conventional assessment framework [33,34]. Specifically, there are no established indicators or measures reflective of the multi-scaled (e.g., familial, community level), intricate connections between people, nature and the spirit world that many Indigenous people across the United States and beyond consider integral to health and wellbeing and life itself (*cf.* [25,26]).

#### 2.1.2. Approach

The Swinomish partnered with the Lower Elwha Klallam Tribe, the Port Gamble S’Klallam Tribe, the Suquamish Tribe and the Stillaguamish Tribe. Tribal representatives expanded a preliminary set of indicators that Swinomish had begun during a previous STAR project (“Bioaccumulative Toxics in Native American Shellfish”, RD#82946701, 2002–2006; see [27]). Tribal reps gathered data from multiple sources to inform the process: interviews with tribal members and experts, literature reviews, and ethnographic records. The collaborative result is a set of six Indigenous Health Indicators (IHIs) that reflect key health considerations often absent in public health assessments, but essential to the Coast Salish way of life: community connection, natural resources security, education, cultural use and practices, self-determination and emotional stability, each with specific attributes and measures [35,36].

To pilot test the indicators, each tribe held a facilitated workshop with tribal members, which tested the clarity, accuracy, and relevance of the IHIs. Researchers used PowerPoint with Turning Point polling software to collect data and display results. The polling software tallies answers via wireless, hand-held polling devices, quickly collates simple statistics, and visually depicts answers in the PowerPoint presentation (e.g., bar graph or pie chart). Using the polling devices allows individual responses to remain anonymous in a room full of familiar faces and the rapid representation of results spurs further discussion.

Each workshop consisted of 12–20 participants from 20–79 years old, from a variety of professions and ways of life. The first set of questions was demographic. The second set of questions established a baseline snapshot of the current community health in relation to the six indicators by answering a series of ranking questions using the IHIs. Each indicator was ranked on a four-point descriptive scale (*i.e.*, very bad to great). In the third section, questions focused on ranking the attributes used to describe each indicator. In a fourth and final question set, participants ranked and weighted (using swing weighting techniques) the IHIs, considering which would be most important to address first during two hypothetical yet realistic scenarios of local pollution events. 

Three criteria were established prior to the workshops to help gauge the accuracy and applicability of the IHIs:

(1) Do the rankings/weightings make sense in terms of expressing accurately what participants feel is important?
(2) Are there distinctions among participants in each workshop in terms of how the indicators were ranked (an assumption of equality between the IHIs would not present an accurate picture of the views of community members)?
(3) Are there distinctions between the two hypothetical scenarios in each workshop (an identical result for the two scenarios would not demonstrate sufficient sensitivity in the measures)?

Based on the results and participants’ positive feedback, the IHI trials met the three criteria and were successful [35]. Each tribe’s workshop results were presented to their respective council for review. Verbal presentations provided each council with a summary of the results as well as some potential uses for the IHIs: establishing baseline community public health status, use in emergency preparedness planning, setting cleanup guidelines, and a host of other health-related policies, both on and off reservation, with the caveat that the indicator set is still in the testing phase. Feedback from the councils has been supportive, with ongoing discussions about how the IHIs can be further refined and utilized into decision-making practices and policies. 

For any kind of health assessment to accomplish its purpose, it must be based on a group’s beliefs and values—*i.e.*, what is valued, what may be at risk and how it may be impacted [37]. Inclusion of the group’s knowledge, and the values originating from that knowledge, must be an equitable part of the decision-making process [38,39,40], yet still situated within the political state. Incorporating indigenous knowledge with other knowledge systems has been problematic historically. Taken out of context, or inserted into a western science framework, indigenous knowledge can be misrepresented, misunderstood, or both (*cf.* [41,42,43]). Project researchers are therefore cautious with respect to the proposed IHI framework. By making use of constructed scales [44] to describe some of the key nonphysical, community-based environmental indicators of Indigenous health, the long-term goal is to provide an equal playing field for learning more, from both Western and indigenous perspectives, about how past, present and future changes to the natural resource base can affect Indigenous environmental public health, as defined by the people themselves.

The primary result of this project is a pilot-tested evaluation tool—the Indigenous Health Indicator (IHI) set—that brings facets of health and wellbeing, as defined by the communities themselves, front and center in the discussions of how to assess and reduce environmental health disparities. Using a tool such as the IHIs provides a more complete and accurate evaluation of health, what is at risk and why, which improves cultural relevancy and provides results for crafting more effective interventions. Based on the pilot-test results, the IHIs reflect widely prioritized aspects of health in Coast Salish communities. The six indicators resonated strongly in each of the Coast Salish communities that pilot-tested the indicators. Workshop results demonstrated that the constructed descriptive scales used to evaluate the indicators met the core criteria necessary to be a successful metric: relevant, useful, understandable, sensitive to change, and reflective of unique considerations (not redundant) (*cf.* [45]). Thus far, several potential uses of the IHIs have been identified for improving: human health risk assessments, health impact assessments, natural resource damage assessments, measuring baseline community environmental health and setting goals, and ecosystem services evaluations. In addition, the measures for each of the indicators can be tailored to fit individual community’s health beliefs and priorities. Swinomish researchers are currently partnering with tribes across the country to test the efficacy of the IHIs in a range of diverse communities, and welcome enquires in regards to the IHIs and potential collaborations. 

The STAR research funding allowed the tribe to increase internal research capacity and understanding of the topics such that the tribe continues to lead the research focus by writing and enacting subsequent studies. Projects continuing the IHI work are underway; one recently completed exploratory trial used the IHIs to evaluate community health impacts from sea level rise with two Coast Salish communities [7]. In June 2014, Swinomish received a new STAR grant to operationalize the IHIs in climate change planning for the Swinomish Tribe. The Tribe plans to study how the combination of sea level rise, wave impacts, and shoreline development will change coastal ecosystems that support Swinomish first foods and place-based relationships, which in turn impact community health and well-being (“Coastal Climate Impacts to First Foods, Cultural Sites, and Tribal Community Health and Well-being,” #RD83559501, 2014–2017, PI: Donatuto). Results will be incorporated in planning and decision-making as part of the Swinomish Climate Change Impact Assessment and Action Plan [46,47].

### 2.2. Apsaalooke (Crow Tribe)

PI and Contributors: Anne Camper, PI for the EPA and NIMHD-NIH sub-awards, Montana State University (MSU); John Doyle, Senior Researcher and Crow Tribal member, Little Big Horn College (LBHC); Margaret Eggers, EPA Star Fellow and PI for NIMHD and NIGMS sub-awards, LBHC; Tim Ford, PI for EPA STAR grant, University of New England and initial PI, NIMHD-NIH subaward, MSU.

The Crow Reservation in south-central Montana is rich in water resources, and water has always been a source of health for the Crow people. However, over the past 50 years, intensifying agriculture and the installation of home wells into shallow groundwater has generated widespread community concern about health impacts from contaminated water sources. These concerns led tribal members and a local tribal college faculty member to conduct a community-wide environmental health assessment. Among the many issues identified, the group prioritized well and river water contamination as their top environmental health concern for the tribal community. Based on this consensus, the project team formed the Crow Environmental Health Steering Committee (CEHSC) in 2005, consisting of representatives of Little Big Horn College (the Crow Tribal college), the Apsaalooke (Crow) Water and Wastewater Authority, the Crow Tribal Environmental Protection program and other Tribal offices, the Crow/Northern Cheyenne Indian Health Service Hospital, and Messengers for Health (a local non-profit), as well as Crow Elders and academic partners from Montana State University Bozeman (MSU). With the exception of the two non-voting partners from MSU, all members of the CEHSC and the project staff are Crow tribal members. This team has been working together ever since to research, communicate and mitigate local water quality and human health issues. The CEHSC meets monthly, about 10 times per year, to set priorities and to plan, guide, participate in and disseminate this work.

#### Approach

With partners Little Big Horn College (LBHC, the local Tribal College) and Montana State University Bozeman, the Crow Environmental Health Steering Committee (CEHSC) conducted a community-engaged, cumulative risk assessment of exposure to waterborne contaminants on the Reservation (EPA RD#83370601-0, 2009–2014; EPA STAR Fellowship FP#91674401; sub-awards of P20MD002317, NIMHD-NIH, and P20GM103474, INBRE, NIGMS-NIH). The Crow Project Coordinator and LBHC science majors collected and tested home well water samples to collect data on well water contaminant concentrations, and conducted surveys on homeowner uses of well water. Key informant interviews, carried out by Crow tribal members, documented family strategies for coping with poor quality well water [48]. Uranium, manganese and nitrate were found to be the inorganic contaminants most frequently exceeding US EPA standards. More than half the wells tested exceeded one or more EPA standards for inorganic and/or microbial contamination [49]. Well test results were reported back and explained to the 150+ participating families both in print and in person. Community education on well water contamination has been conducted through presentations at and discussions with the CEHSC, the Crow Water Resources Department staff, local groups, open houses at LBHC and community health fairs. Local schoolchildren are being reached through classroom visits and teacher trainings (with continuing education credits) [12]. Well testing as well as community education and outreach on the health effects of well water contamination are ongoing.

Mitigating unsafe well water is a challenge. Project survey results showed that only 4% of families had a reverse osmosis system, although 85% of wells exceed the EPA standard for TDS [12]. The high cost of purchasing, installing and maintaining a cistern or traditional water treatment technology is prohibitive for many families; the per capita income in Crow communities on the reservation averaged from $7354–$8130 in 2010, about 1/3 of the Montana per capita income of $23,836 [50]. Recent follow up visits with the 30 families consuming well water with the highest levels of inorganic contaminants found that all of the families are now hauling better water for drinking and two thirds have switched to hauled water for cooking. A low cost, high tech home water filtration system for families with poor well water has been pilot tested by Crow families [51]. That system is now being redesigned as a result of community feedback, and a second round of pilot testing is being considered for the fall of 2015. Lack of access to safe drinking water is an environmental health disparity [12]. 

Baseline testing of rivers found that river water was unsafe for direct consumption year round, in all locations, due to microbial contamination. During spring runoff and in late summer in some locations, fecal contamination exceeds the limit even for safe recreational uses [52]. As many tribal members maintain traditional ceremonial practices that include drinking river water untreated and using it for bathing [48], and children swim in the rivers summer long, the documented fecal contamination levels create a substantial public health risk. Interviews with 30 key informants found that all but one interviewee were now aware of microbial contamination, and many had given up recreational swimming—however, those families who maintain ceremonial practices involving the rivers had not given these up, because the traditions “are what makes us Crow” [48]. Yet, as there is no public pool available on the reservation, children continue to use the swimming holes through the hot summer months. The project team subsequently wanted to initiate an environmental health literacy campaign on water quality and stewardship with local school children; this project has been funded and is now in the planning stages under the leadership of Dr. Vanessa Simonds, a Crow tribal member now a member of the faculty at MSU Bozeman.

Additionally, the research data have supported the Apsaalooke (Crow) Water and Wastewater Authority in bringing in more than $20 million to upgrade and replace failing municipal water and wastewater infrastructure. This funding allowed for the replacement of the century-old Crow Agency wastewater lagoon as well as miles of pipes in the equally ancient, leaking distribution system. These renovations have improved the quality of the municipal water being delivered to people’s homes, as well as the quality of the treated wastewater being returned to the river [53,54]. Wastewater no longer backs up into people’s homes nor surfaces in puddles throughout town. What began as an effort to address municipal water contamination in one community has expanded into many other areas, in unanticipated ways. For instance, the project team has begun researching the current and projected impacts of climate change on reservation water resources and community health, drawing on both community environmental knowledge and western science. The team found that meteorological data confirmed community observations of declining annual snowfall as well as increases in frost free days and in hotter summer days. Community knowledge contributed additional insights about local ecology not documented by Western science, such as changes in fish species distributions and in plant growth and phenology. In short, meteorological data and community environmental expertise both support and complement each other in understanding local impacts of climate change [8]; this research as well as adaptation planning is being continued through a new 2014 EPA STAR grant.

The project team has been seeking to identify the source(s) of and mitigate the fecal contamination of a culturally vital spring in the community of Pryor on the reservation, utilizing microbial source tracking and isotope analysis techniques with new collaborators. Co-author Doyle meets regularly with the Pryor Elders Committee, who are helping to identify potential contamination sources and sampling sites, and appreciate the opportunity to hear about and discuss project progress. Until the issue can be resolved, a sign has been posted by the spring notifying people of the microbial contamination.

Community capacity in environmental and health disciplines is increasing. Four Crow former project team members, including two student participants, have gone on to earn Master’s degrees. MSU team members secured graduate funding for minority students in health disciplines, which supported these two students and is now funding two additional Crow tribal members in obtaining Master’s degrees in health disciplines. Several other former participants have now earned bachelor’s degrees in science disciplines and are back working for the tribe, the Bureau of Indian Affairs or are teaching at Little Big Horn College. 

The most significant impact of the *Apsaalooke (Crow Tribe)* research is the initiation of dialogue on water quality in the Crow reservation community: about what it will take to clean up the rivers and to cope with well water contamination from both natural and anthropogenic sources. These conversations were simply not happening before. Homeowners with poor quality well water had previously been concerned with the smell, taste and discoloration of their well water, but had not realized that inorganic contaminants such as uranium could be present and could increase the risks of serious health effects. Many homeowners who learned that their well water was unsafe to drink due to microbial contamination were able to get help from the Crow Tribal Environmental Protection Department to shock chlorinate their well. Others who learned their well water had inorganic contaminants exceeding EPA drinking water standards have sought out cleaner sources of water and are spending what they can afford on purchasing better water for their families. Collectively, exposures to microbial and inorganic contaminants via well water have been reduced. Standard treatment technologies such as water softeners and reverse osmosis units remain prohibitively expensive for many families. There is greatly increased interest among community members in any future opportunity for free well water testing.

One of the team’s ongoing goals is to share experience and research with other tribes, for instance, by presenting at and learning from others at the National Congress of American Indians Tribal Leader/Scholar Forums [53,54] and EPA Tribal Science Forums [55]. It is important to build connections with one another to share what works and what does not, recognizing that what is true for one location may not be true for another. Through creating and strengthening connections, tribal communities can build capacity, save cost and improve health and wellness. The team hopes that everyone’s collective work will continue to grow and will bring more to native communities wherever they are. The team invites readers to contact them.

What began as an effort to address municipal water contamination in one community has expanded into many other areas, in unanticipated ways. The topic has always been water, and what began as a little trickle has grown into a stream. Through these projects, the CEHSC has found that they can successfully tackle problems, identify suitable research partners, effectively collaborate with partners on mutually acceptable terms, secure funding and present and publish results [8,52,56,57,58]. This process requires commitment, passion, mutual support, hard work and persistence [56]. Community capacity to conduct research has increased: for instance, initial grants were awarded to MSU with sub-awards to LBHC; the current grant is awarded to LBHC with a sub-award to MSU, with a founding CEHSC member who is Crow as the LBHC Co-PI. The project team is still learning about water and health and how to facilitate change, yet now as a result of the above described experiences and projects, feels empowered to work to improve the well-being of our Native community. 

### 2.3. Alaska Native Tribal Communities and Wild Berry Resources (PI and Contributor: Mary Ann Lila)

#### 2.3.1. Background

The EPA STAR Project (EPA R833707, Impacts of climate change on health benefits of a tribal Alaskan resource: Integrating traditional ecological knowledge with risk assessment through local monitoring, 2008–2011), engaged social and biological sciences research teams from the University of Illinois and Rutgers University with naturopathic medicine professionals at the Alaska Native Tribal Health Consortium and elders and youth from three Alaska Native (AN) communities: Seldovia (on the Kenai peninsula), Akutan (in the Aleutian Islands), and Point Hope (north of the Arctic Circle). The primary objectives of the initiative were to simultaneously: (1) conduct integrated research on how climatic stress factors might influence the health-protective properties of wild Alaskan berries (a subsistence wildcrafted crop in many Native communities, used for food and medicine); and to (2) assess local traditional ecological knowledge (TEK) and risk perceptions regarding these berries, given the seasonal shifts associated with climate change. Because of the dual biological/social sciences foci of this project, and in response to the dialogue of concerns repeatedly voiced by elders involved in this project, the project soon was informally nick-named “Alaska berries and human health, under the cloud of climate change” [59].

The health disparity targeted in this project was the high incidence of diabetes/metabolic syndrome in AN communities, and the recognized capacity for dietary berries to combat incidence of these disease conditions. For many communities (in particular, in the far north), minimal land for gardening was available around the villages. Wild berries are almost the exclusive terrestrial edible plant used for food and medicine in TEK. Due to the harsh climate endured by arctic wild berries, these wild berry species (salmonberries, cloudberries, lowbush cranberries, bog blueberries, mossberries, and more) accumulate intense profiles of phytoactive chemicals that are highly immunoprotective, and account for their remarkable health-protective properties [59,60,61]. Any other fruit (or vegetable produce) must be shipped in from the lower 48; availability is scarce and costs are exorbitant. In part, due to loss of traditional practices and a reluctance among AN youth to recognize the health properties of the wild berries, the incidence of type 2 diabetes mellitus has escalated in the communities. The innovation provided by the project was a mixed methods approach that integrated biological sciences (to make quantitative measurements of the bioactivity and health-relevant value of wild berries in AK), and social sciences approaches to partner elders and traditional knowledge with the science activities. 

#### 2.3.2. Approach

The team used a unique “Screens to Nature” (STN) strategy to gauge health-protective properties. The STN workshops partnered university researchers with elders and youth, and local school teachers from each community using simple field deployable bioassays to assess bioactive mechanisms and properties associated with the wild fruits This tactic brought youth and elders together, and particularly pleased many of the elders in that the youth participated in obtaining bioscience results, which clearly and unambiguously corroborated the wisdom of local TEK; for example, plants that were cited in TEK as useful remedies for high blood sugar were subsequently proven in the bioassays to regulate alpha-glucosidase or alpha-amylase activity [59,61]. The STN approach opened previously unapproachable avenues for research, in that the communities were aware that all resources stayed in the community, and any potential intellectual property that may arise from an STN discovery remained in the hands of the communities. The STN workshops were coupled with youth-led community surveys, interviews and information-sharing sessions, which revealed that students were taking more active interest in traditional foods which had an impact on diabetes and other diseases. These outcomes sparked interest among local teachers for using the bioassays and approach as a teaching tool in their own classrooms year round, to incentivize youth towards science by using native resources as the experimental subjects [62,63]. In some communities, the science-based results provoked interest in potential for commercialization of the wild resources, whereas in other communities, the village councils decided not to seek out commercial opportunities [64]. 

In all cases, the relationships forged in the EPA STAR sponsored outreach led to sustained, continuing partnerships with AN communities, generated new opportunities for interaction (for example, a current USDA HEC-sponsored education-focused project with University of Alaska Fairbanks and University of Alaska Anchorage), and has branched out into expanded bioexplorations and STN validations of not just wild berries, but also of marine resources like local seaweeds used for foods and medicines [65]. Concerns about climate change in arctic communities, and how environmental changes will possibly alter the health-protective attributes of native plants, can only be fully addressed with multiple year assessments in the affected communities. The project’s results have been picked up by local press (Alaska Dispatch) and continue to be used and reported in multiple forums, such as during the annual Alaska Plants as Foods and Medicine conferences. These reports coupled with testimonies made by local teachers in AK native communities have prompted repeated requests to host STN workshops in other AN communities; our team and our AN partners continue to pursue funding opportunities to allow expansion of the outreach. 

In the *Alaska Native Tribal Communities and Wild Berry Resources* project, direct and indirect impacts of climate on wild berry phytoactive chemical composition and biological activity against diabetes were also documented. Due to the short duration of the project, true effects of climate change could not be gauged, but the research results illustrated that berries growing in the northernmost extremes of Alaska accumulated a greater concentration of bioactive phytochemicals than berries growing in more moderate climates. This observation gave indication that moderating temperatures (as could occur during climate change) may cause subtle changes in the health protective properties of this native resource. The research outcomes provided AN elders and youth with a greater appreciation of how their climate contributed to production of a unique and exceptionally valuable local resources—native berries—especially as the data compared local berries to categories of other berries produced in the lower 48 states of the USA as well as South America. Not only were the community members informed with the direct findings in the research, but they were empowered with field-deployable bioassay kits that could be used by community-based schools for years to come, so that comparisons in bioactive potential could take place over a number of years and climate scenarios. Elders who participated in the project commented that “we always knew that our weather and the stresses made the berries tastier and heartier, and it is good to see they are healthier too”.

### 2.4. Traditional Food Safety in a Warming Climate: A Brief Case Study Describing a Tribally-Designed Yupik Alaska Native Maternal Biomonitoring Program to Assess Food Safety and Facilitate Development of an Adaptive Strategy (PI and Contributor: James Berner)

#### 2.4.1. Background

World-wide production and use in agriculture and industry of organohalogen (OH) compounds over the last 70 years has resulted in environmental dissemination by atmospheric, river, and ocean currents, with uptake by biota in every marine and terrestrial ecosystem, including the Arctic. In general, these compounds are lipophilic, and persistent in fat tissue over decades, giving long exposure to organisms, and, especially in mammals, are efficiently transferred to their offspring during lactation. These compounds were discovered in the breast milk of Canadian Inuit mothers' breast milk in the 1980s. This information eventually resulted in the international action of the eight nations with territory in the Arctic to form the international organization that was later named the Arctic Council (AC). Among the first actions of the AC was the creation of the Arctic Monitoring and Assessment Program (AMAP), to measure levels of OH and heavy metals (HM) in environmental matrices and biota, including human residents. A few years later, tissue trends and health effects were added to the AMAP responsibilities. Representatives with responsibilities in human health, toxicology, and environmental health were designated by each country, and formed the AMAP Human Health Assessment Group (HHAG). The HHAG designed a basic maternal blood biomonitoring program, designed to sample Arctic women who delivered at local hospitals in regions with residents who consumed a diet with marine fish and sea mammals. Blood was taken from women who agreed to participate on entry at delivery, and umbilical cord blood was obtained from the infant at delivery. 

The first report of the OH and heavy metal blood levels were published in 1998, and contained no human data from Alaska, as no federally initiated maternal monitoring program existed. When Alaska Native (AN) leaders learned of the results of the AMAP monitoring program in the other Arctic countries, they began active planning for their own program. AN Tribal health organizations viewed the 1998 AMAP report as raising the issue of the safety of their traditional subsistence food. The subsistence diet is a central component of all AN cultures, it is economically critical to rural AN communities which are isolated, very small, and are often economically dependent on their traditional diet to supply a substantial part of their protein and micronutrient intake. The northern marine subsistence diet also has been shown to have considerable population health benefits not found in many common Western replacement food sources. 

#### 2.4.2. Approach

The EPA has long recognized the need to better understand transboundary pollution issues, and in the last decade of the 20th century, the EPA Office of International Activities, at the request of the Alaska Native Tribal Health Consortium (ANTHC), entered into an interagency agreement with the Indian Health Service, to fund a maternal blood monitoring program in Alaska. The ANTHC was the tribal agency responsible for working with regional tribal health organizations to take advantage of this opportunity to examine the exposure resulting from subsistence food species accumulating OH and HM contaminants, mostly originating in the Asian river drainage and the rivers of the Russian Far East, into the North Pacific, Bering Sea, and Arctic Ocean. In addition, Alaska Natives wanted more information about possible human health effects from anthropogenic contaminants, and positive health impacts from the micronutrient components of traditional subsistence species. Initial exposure data indicated AN mothers had an exposure similar to women in Scandinavia, Iceland and western Canadian Arctic coast communities. Numbers of enrolled women were not sufficient to examine the data for significant associations with existing health disparities.

The Alaska Maternal Organics Monitoring Study: An Epidemiologic Study of Cumulative Health Effects of Persistent Organic Pollutants and Mercury in Subsistence-Dependent Rural Alaska Natives, was a Tribally-designed Maternal Organics Monitoring (MOM) Study protocol that measured all the analytes used in the AMAP protocol, but also measured micronutrients such as omega-3 fatty acids and selenium, aimed at determining the potential benefits and positive health effects, as well as any adverse impacts associated with the OH and HM found in subsistence food. In addition, women were enrolled at the first prenatal visit, so that events in early pregnancy could be analyzed, and examined for association with any analytes. A short dietary survey was completed by participants during their hospitalization for delivery, focused on the subsistence foods during their pregnancy. Medical record reviews were performed on mothers at least six weeks after delivery, and infant charts were reviewed after 12 months of life, and information obtained was entered in a database populated with levels of the OH and HM compounds analyzed in the AMAP protocol tested.

A unique component of the AN biomonitoring program was the decision to include study of the most common subsistence species consumed by this population, the Pacific salmon that are harvested yearly from the Yukon and Kuskokwim Rivers that form the Yukon-Kuskokwim Delta, the region inhabited by the Yupik AN people. Two samples of salmon from these rivers were compared, from 2001 and 2010. These fish spend their pelagic life cycle in the Bering Sea and North Pacific Ocean, and are reflective of the seawater concentrations of the OH and HM in the subsistence food chain. 

The AN people of the Yukon-Kuskokwim Delta are among the most subsistence-dependent U.S. residents, due to their remote location, and lack of an economical alternative to their traditional diet. For the most part, they also prefer the subsistence species to western foods, although Western foods are becoming more available, although very expensive. Management of the harvest of sea mammals as a subsistence species is managed by a Co-management Commission, composed of federal wildlife managers, and Alaska Natives, thus avoiding the tension over extent and scope of the harvest. Only Native Americans are allowed, by federal law, to harvest sea mammals, and this system of joint management has worked remarkably well. The analysis of village location (coastal compared to river) of the maternal participants in the biomonitoring program showed an association between Mercury blood levels and coastal residence. The levels were similar to other circumpolar maternal populations, but review with tribal communities showed that they were aware that younger, smaller sea mammals had generally lower levels of contaminants and mercury, and that land mammals had very low, often undetectable levels. Data on hunting and harvest data is still being analyzed, but the early trend data in maternal blood levels shows a decrease across all contaminant and heavy metal categories. If the harvest data supports the hypothesis that this is due to a change in harvest practices, or maternal consumption patterns, this would support the hypothesis that an adaptation strategy by village hunters and pregnant residents has taken place, likely in response to the study data.

A total of 502 Yupik women have been enrolled in the MOM Study since 1999, which has been supported by the EPA and CDC and the AN Tribal health organizations, especially the Yukon Kuskokwim Health Corporation, and the Alaska Native Tribal Health Consortium. Analysis of the laboratory data is not yet complete, and analysis of health effects cannot be completed until the final laboratory results are obtained. 

The EPA STAR Project (EPA R833705, *An Epidemiologic Study of Time Trends and Health Effects of Persistent Organic Pollutants, Mercury and Micronutrients*, 2009–2014) offered the opportunity to extend the maternal biomonitoring program with an additional 160 women and infants, and to, re-sample key salmon species for OH and HM levels. This project specifically addressed cumulative exposure to multiple environmental stressors in rural Yupik Alaska Natives (AN), by investigating anthropogenic persistent organic pollutants (POPs) and mercury (Hg) as stressors that accumulate in pregnant Yupik residents through ingestion of traditional subsistence wildlife species, particularly salmon and seals.

The *Traditional Food Safety in a Warming Climate* project demonstrates that a carefully designed human biomonitoring program can be created and implemented by a tribal organization, and can be designed to contribute valuable data for federal agencies concerned with the control of production and release of OH chemicals, and HM. This creates a mutually supportive relationship, with agencies contributing support for organizations able to collect data that the agencies would otherwise not be able to access. When the data is complete, this research can potentially contribute exposure and effects data to the field of mixture toxicology, nutrition and public health, as well as allow the development of community adaptation strategies in subsistence harvest that will lower exposure to OH and HM in pregnant residents. This study demonstrates the utility and adaptability to conduct the basic AMAP maternal biomonitoring model for most subsistence dependent communities in any region.

## 3. A Decade of Tribal Environmental Health Research: Results and Impacts from EPA’s Extramural Grants and Fellowship Programs

In January 2014, NCER released a report titled “A Decade of Tribal Environmental Health Research: Results and Impacts from EPA’s Extramural Grants and Fellowships Programs”, summarizing over a decade of Tribal Environmental Health Research [66]. Community-based research conducted by the grantees and their partners have assisted tribal communities to identify solutions and apply interventions that have reduced health and ecological effects from the consumption of water and water-based food resources, chemical contaminants, and impacts of climate change while enhancing the ability to conduct community-level risk assessments. STAR grants strive to support tribal citizens’ cultural practices while reducing health risks. These projects also help to strengthen Native language skills and increase culturally relevant communication of traditional ecological knowledge. AI/AN communities often follow traditional diets that include an abundance of freshwater fish and seafood. Water, considered sacred, plays an important role in tribal cultural and spiritual practices. Several STAR grants (including several of the case studies presented here) focused on reducing the health effects associated with the consumption of contaminated traditional subsistence foods and water resources. This is one example of how tribal research has led to the practical use of data on contaminant levels to help community members protect their health while following their traditional diets. Using CBPR in tribal research ensures that AIAN populations have a voice. CBPR and other aspects of community engaged research e.g. community outreach and education, continue as longstanding, important components of STAR grants and fellowships funded under the Tribal Environmental Health Research Program. Most of the grants use community outreach and tribal consultations to obtain input that guides the research projects. Tribal citizens learn about the results of the grants through community presentations, training and workshops, books, DVDs, maps, radio interviews and other means. Based on STAR results, researchers produced education and outreach pieces such as a traditional foods and harvesting practices book, a children’s traditional foods coloring book, and a documentary, “Slow Burn”, about contamination of local natural resources [67]. 

Overall, these grants yielded data, tools, products, methods and knowledge that have helped AIAN communities to better define and reduce their health risks, protect natural resources essential to cultural and spiritual practices, and encouraged the ecological knowledge and tribal practices of protecting and preserving the earth for future generations.

As shown in the case studies above, federal support of tribal community-based research is an important infrastructure through which burdened and resource-limited communities can (1) obtain reliable and flexible funding; (2) develop or gain access to a cadre of technical and academic tools (on site or through collaborative partners) to determine and prioritize problems to be explored; (3) build and strengthen community capacity for addressing research and development needs; and (4) secure a safe and supportive environment to collaborate on or initiate research projects, develop culturally relevant tools, risk modeling tools, and focused public health messaging. An added benefit of research conducted in these settings is the identification of critical data, methodologies, and recommendations that can be utilized for decision making, and influencing policies and regulations on the tribal, local and state levels.

## 4. Conclusions and Recommendations

**Figure 1:**
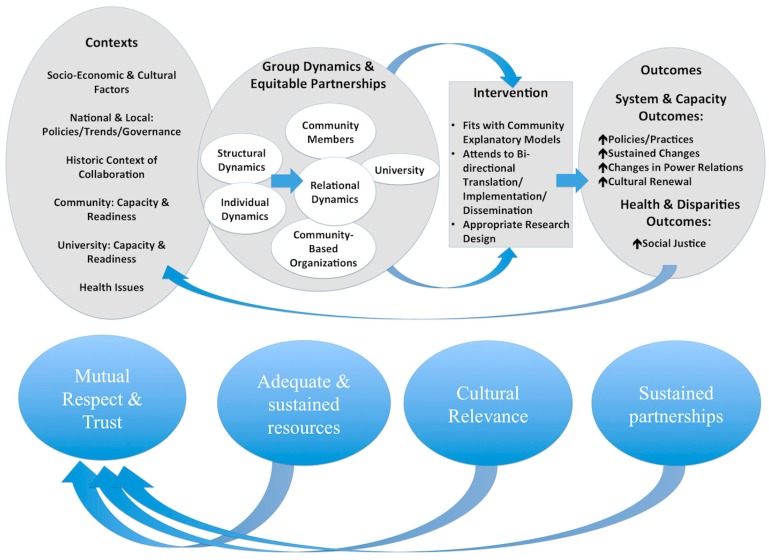
Case study conceptual CBPR model as an example (Figure redrawn and adapted from [31], with permission).

The four key conditions highlighted in the case studies—cultural relevance, mutual respect and trust, adequate and sustained resources, and sustainable partnerships—are inter-related and also linked to broader CBPR concepts. Figure 1 depicts a conceptual model of these relationships between the four key conditions and CBPR; the conceptual model is based on modifications to the Wallerstein *et al.* model [31,32], to which the National Council of American Indians Policy Research Center contributed.

Two of the most important steps for moving forward in tribally-engaged and *tribally-driven* research are: sustained approaches that can sustain long term capacity of AIAN communities, and increased capacity for AIAN communities to partner and share knowledge with other communities, empowering tribes to carry out the work themselves. In regards to the first step, many if not all of the STAR projects have successfully identified data gaps or much needed second phases of the research. Future efforts will require that AIAN community members and their research partners and collaborators develop strategies and approaches to ensure success and longevity of research endeavors. AIAN communities facing environmental health disparities will continue to strive to reduce adverse health and ecological impacts in their communities by identifying and prioritizing critical research needs, and through commitments and combined efforts from research partners, achieve culturally meaningful and sustained translational outcomes.

The second step is to enhance knowledge sharing networks and collaborations between and among AIAN communities in order to bolster AIAN-to-AIAN information sharing on research design, implementation and results. Such networks already exist formally (e.g., via the National Congress of American Indians, regional and national caucuses) and informally (via colleagues and relatives), but there is no well-known and centralized location for such knowledge that has forums for discussions and contact information for additional details. The goal of such a network would be, ultimately, sharing information to empower AIAN communities to enact their own prioritized research themselves, building on the knowledge and assistance of relatives and colleagues. 

The impacts of environmental contamination on environmental health when communities maintain traditional, spiritual relationships with the natural world (animals, plants, water, *etc.*) and related subsistence practices need to be valued and accounted for not only in local environmental health risk assessments [35,36], but also in regional EJ mapping tools, such as EPA’s EJView [68] and California’s EJ Screening Methodology [69], and accounted for in federal programs. The incorporation of local tribal-specific definitions of health or at a minimum giving weight to maintenance of subsistence practices and traditional relationships with the environment could help ensure equitable environmental regulation and allocation of federal and state resources for mitigation. In the case of U.S. federally recognized tribes, treaty-secured rights ensure protections of health for tribes and for their natural resources [70]. 

To help build research capacity in AIAN communities, the best practices, methods and lessons learned from EPA STAR fellowships, which provide support for advanced studies in environmental health for AIAN and other qualified students, (e.g., the Greater Opportunities (GRO) and STAR graduate fellowships, see [71], and research grants can be disseminated and integrated into informational and educational materials, publications and other relevant materials for use by the general public, researchers and decision makers. 

Research capacity could also be expanded and strengthened for stakeholders interested in protecting their community’s health and environment. For example, programs and curricula can be expanded or developed for students to pursue a graduate degree in environmental health, with a focus on helping their communities through community-engaged research. A graduate scholarship model has already been demonstrated to work (e.g., “Improving Montana Community Health through Graduate Education”, NIMHD-NIH #1R25MD006791-01, 2011-2015, PI: Anne Camper). Further, “community research capacity building” could be considered as an additional evaluation criteria for research proposals. 

The case studies presented in this article demonstrate tremendous creativity and power in the design and implementation of these *tribally-driven* research and mitigation projects, growing out of the rich collaborations characteristic of fully community-engaged partnerships among tribes, academia, health care providers and others. These case studies illustrate that when tribes identify the priority issues to be addressed, drive the research design, implementation and dissemination of results, are meaningfully engaged throughout the process when they are partnering with academic or other institutions, and have sustained partnerships and funding, then CBPR is effective in addressing environmental health disparities in tribal communities.
